# Rab11b is necessary for mitochondrial integrity and function in gut epithelial cells

**DOI:** 10.3389/fcell.2025.1498902

**Published:** 2025-04-03

**Authors:** Ivor Joseph, Jiangmeng Han, Jared Bianchi-Smak, Jiaxing Yang, Jagannatham Naidu Bhupana, Juan Flores, Jack Delucia, Tracy S. Tran, James R. Goldenring, Edward M. Bonder, Nan Gao

**Affiliations:** ^1^ Department of Biological Sciences, Rutgers University, Newark, NJ, United States; ^2^ Department of Pharmacology, Physiology, and Neuroscience, Rutgers New Jersey Medical School, Newark, NJ, United States; ^3^ Department of Surgery, and Cell and Developmental Biology, Vanderbilt University School of Medicine, Nashville, TN, United States

**Keywords:** Rab11, mitochondria, Paneth cell, proteomics, intestine

## Abstract

**Introduction:**

The RAB11 family of small GTPases are intracellular regulators of membrane and vesicular trafficking. We recently reported that RAB11A and RAB11B redundantly regulate spindle dynamics in dividing gut epithelial cells. However, in contrast to the well-studied RAB11A functions in transporting proteins and lipids through recycling endosomes, the distinct function of RAB11B is less clear.

**Methods and Results:**

Our proteomic analysis of RAB11A or RAB11B interactome suggested a potential RAB11B specific involvement in regulating mitochondrial functions. Transcriptomic analysis of Rab11b knockout mouse intestines revealed an enhanced mitochondrial protein targeting program with an altered mitochondrial functional integrity. Flow cytometry assessment of mitochondrial membrane potential and reactive oxygen species production revealed an impaired mitochondrial function in vivo. Electron microscopic analysis demonstrated a particularly severe mitochondrial membrane defect in Paneth cells.

**Conclusion:**

These genetic and functional data link RAB11B to mitochondrial structural and functional maintenance for the first time.

## Introduction

Epithelial cells of the mammalian small intestine form a dynamic tissue layer that separates the host from the luminal stressors. This monolayer of epithelial cells is composed of the finger-like villi associated with the cup-shaped crypts of Lieberkühn. Villus epithelia are primarily composed of mature, polarized absorptive enterocytes with secretary goblet and enterocrine cell types scattered along the monolayer. The crypt compartments harbor actively dividing stem cells and terminally differentiated Paneth cells at the base of each crypt, and transit amplifying cells residing at crypt-villus junctions (Sumigray et al., 2018; Clevers, 2013; Tan and Barker, 2014). The gut epithelial compartment is evolutionarily designed for massive reproduction and replenishment of cells, as the entire intestinal epithelial sheet renews every 3–5 days.

RAB GTPases play a crucial role in the transport and distribution of proteins to specific cellular compartments (Li and Marlin, 2015; Sato et al., 2007). RAB11 protein members are proposed to be the key regulators for the recycling of internalized membrane proteins or lipids back to the plasma membrane in both nonpolarized and polarized epithelial cells (Green et al., 1997; Wang et al., 2000; Hales et al., 2002; Joseph et al., 2023; Kitano et al., 2021; Sobajima et al., 2014; Sobajima et al., 2018). In *Drosophila*, RAB11 regulates the cell surface proteome and facilitates integrin β1 trafficking for efficient extracellular matrix engagement in the brain promoting cell survival (Howe et al., 2020). The mammalian RAB11 family includes three isoforms: RAB11A, RAB11B, and RAB25 (Bhartur et al., 2000; Kumar and Lukman, 2018). They are abundantly expressed in polarized cells and localizes to subapical recycling endosome in MDCK and Caco-2 cells (Wang et al., 2000). The subapical recycling endosome in Caco-2 cells showed little overlap with TfR-positive basolateral early endosomes (Prekeris et al., 2000). RAB11A and RAB11B share 89% sequence identity and exhibit some overlapping functions, while RAB25 is more divergent (Bhartur et al., 2000; Kumar and Lukman, 2018). RAB11A was originally isolated from bovine brain tissue (Kikuchi et al., 1988; Sakurada et al., 1991; Bhuin and Roy, 2015), and was subsequently found to be ubiquitously expressed in mammalian cells (Bhuin and Roy, 2015; Xu et al., 2011). RAB11A localizes specifically to the recycling endosome linking its function to regulation of protein and lipid trafficking from the recycling endosome to the plasma membrane (Ferro et al., 2021; Maxfield and McGraw, 2004; Ullrich et al., 1996). Due to its critical control of anterograde trafficking to plasma membrane, RAB11A influences a wide range of cellular processes, for example, trafficking of transferrin to the plasma membrane (Green et al., 1997; Ullrich et al., 1996; Ren et al., 1998; Schlierf et al., 2000) for iron transport and uptake (Gkouvatsos et al., 2012), and trafficking of E-cadherin, H^+^/K^+^-ATPases, CFTR, ENaC, TRPV5, and TRPV6 for epithelial cell polarity establishment and electrolyte transport (Desclozeaux et al., 2008; Welz et al., 2014; Calhoun and Goldenring, 1997; Calhoun et al., 1998; Berger et al., 1991; Silvis et al., 2009; Karpushev et al., 2008; van de Graaf et al., 2006). Furthermore, RAB11A, in conjunction with myosin Vb and RAB11-FIP2, controls the recycling of the AQP2 water channel (Nedvetsky et al., 2007). During cell division, RAB11A supplies essential components to the midbody for cytokinesis (Fielding et al., 2005; Wilson et al., 2005; Schiel et al., 2012). In immune cells, RAB11A regulates trafficking of T-cell receptors (Redpath et al., 2019), autophagy and phagocytosis of apoptotic neutrophils by macrophages (Puri et al., 2018; Jiang et al., 2017), recycling of CXCR2 in chemokine response (Fan et al., 2003; Fan et al., 2004), and distribution of pIgA-pIgR and TLR compartmentalization for mucosal immune responses (Fan et al., 2021; Su et al., 2010; Lapierre et al., 2001; Yu et al., 2014a).

Previously, using genetic mouse models, we reported that global knockout of the *Rab11a* gene in mice resulted in embryonic lethality during the implantation stage (Yu et al., 2014b). Mice with intestinal epithelial cells (IECs) specific knockout of *Rab11a* exhibited multiple epithelial abnormalities including, mislocalization of brush border proteins, increased inflammatory cytokine production, hyperplasia, and elevated susceptibility to tumor development (Sobajima et al., 2014; Yu et al., 2014a; D'Agostino et al., 2019; Feng et al., 2017; Knowles et al., 2015). Additionally, the RAB11A endosome compartment acts as a regulatory platform for Hippo-YAP signaling and epithelial cell proliferation (D'Agostino et al., 2019). Recently, we reported that RAB11A and RAB11B share some redundant functionality in regulating mitotic spindle dynamics in gut progenitor cells, as double knockout mice failed to survive due to loss of capacity for sufficient cellular proliferation to maintain the epithelium (Joseph et al., 2023).

Compared to the extensive knowledge about RAB11A, the cellular functions of RAB11B are largely under-explored and remain unknown. RAB11B was first found to be abundantly expressed in the brain, testis, and heart (Lai et al., 1994), and was found to partially colocalize with recycling endosome, along with cystic fibrosis transmembrane conductance (CFTR) anion channel (Schlierf et al., 2000; Silvis et al., 2009), V-ATPase, and Cav 1.2 channel (Oehlke et al., 2011; Best et al., 2011). However, unlike RAB11A, RAB11B does not colocalize with H^+^/K^+^-ATPases or known RAB11A cargos such as IgA (Lapierre et al., 2003), suggesting different and unique regulatory roles for RAB11B. Cell culture studies suggested that RAB11B may be involved in Ca^2+^ dependent exocytosis of synaptic vesicles (Khvotchev et al., 2003), cysteine protease secretion (Mitra et al., 2007), trafficking cGMP-dependent protein kinase II (Yuasa et al., 2008), recycling PAR1 in HeLa cells (Grimsey et al., 2016), or the exocytosis of insulin granules in pancreatic β-cells (Sugawara et al., 2009). Although our recent studies pointed to RAB11B’s overlapping function with RAB11A (Joseph et al., 2023), few genetic studies have been conducted to determine RAB11B-specific functions in mammalian cell types.

In this report, our proteomic analysis uncovered an association between RAB11B with mitochondrial structural and functional components. Examination of the intestinal epithelial cells by electron microscope revealed that *Rab11b* knockout mouse intestinal epithelial cells have abnormal mitochondrial morphologies. By flow cytometry analysis, we found that epithelial cell from *Rab11b* knockout mice exhibited impaired mitochondrial membrane potential and reactive oxygen species (ROS) production. These observations employing genetic, ultrastructural, proteomic, and transcriptomic studies suggest a previously unappreciated contribution of RAB11B to the maintenance of mitochondrial structure and homeostasis in the gut epithelial cells.

## Materials and methods

### Mice

*Rab11b* ^−/−^ mice were developed by us through CRISPR-CAS9 genome editing previously described in (Joseph et al., 2023; D'Agostino et al., 2019). Mice were housed and bred under a specific pathogen free condition. The comparison of wild type and *Rab11b* ^−/−^ mice were made in both male and female mice, and experiments were repeated using mice from different mating pairs. All animal experiments were conducted in accordance with NIH guidelines and approved by the Institutional Animal Care and Use Committee (IACUC) at Rutgers University.

### Intestinal tissue fixation and processing for transmission electron microscopy (TEM)

The procedure for transmission electron microscopy was described previously (Yu et al., 2014a; Gao and Kaestner, 2010; Balasubramanian et al., 2023; Das et al., 2015; Flores et al., 2021; Yu et al., 2020). Briefly, intestinal tissue samples, approximately 1 mm^3^ in size, were collected and fixed overnight at 4°C in TEM fixative (2.5% Glut and 2.0%PFA in 0.1M Sodium Cacodylate (NaCac) pH 7.4). Following fixation, the samples were washed twice for 10 min each with 0.1M NaCac pH 7.4. The tissue samples were then incubated in 1% OsO4 in 0.1M NaCac pH 7.4 solution for 1 h in the dark. Subsequently, the samples were washed twice with 0.1M NaCac for 10 min each time and then rinsed twice with milli-Q for 10 min each time. The tissue samples were then incubated with 1% Uranyl Acetate in H_2_O for 30 min. After incubation, the samples were washed twice with ddH2O for 10 min each time. The samples were then dehydrated through 50%, 70%, 80%, 90%, and 100% ethanol(2x). The samples were further dehydrated using 1:1 and 3:1 Propylene Oxide:Ethanol and 100% Propylene Oxide for 10 min each. For resin embedding, the samples were left overnight in 1:1 Propylene Oxide: EMBed-812 followed by overnight incubation in 100% EMBed-812. Final embedding was in EMBed-812 activated with DMP at 65°C for 48 h. Ultrathin sections were post-stained using lead citrate and uranyl acetate. Three independent mice were used for each genotype with 3 grids prepared for each mouse. To assess mitochondrial integrity, the morphology of mitochondria was categorized into four grades based on structural and membrane characteristics: normal (intact cristae and preserved membrane integrity), grade 1 (mild abnormalities with minor disruptions in cristae structure), grade 2 (pronounced abnormalities, including swelling and loss of cristae definition), and grade 3 (severe damage with extensive cristae disruption and compromised membrane integrity). Percentage of mitochondria in each grade was calculated for each genotype. The proportion of mitochondrial grades was visualized as a stacking bar graph. The analysis was performed based on a total of 50–70 microscopic images from the 3 mice per genotype.

### RNA extraction, bulk RNA sequencing, and gene set enrichment analysis

RNA extraction and bulk RNA sequencing were performed according to the protocols described in ((Joseph et al., 2023) Total RNA was extracted from 2 to 3 mm sections of jejunum tissue using the Qiagen RNeasy Plus Mini Kit (#74134). Each tissue sample was immersed in 800 μL of RLT lysis buffer (provided by manufacturer) supplemented with 10 μM β-Mercaptoethanol. Tissues were homogenized at 4°C by multiple passages through a 20-gauge needle (BD #305176). Following homogenization, RNA isolation was performed according to Qiagen RNeasy Mini protocol by applying to a spin column and RNA elution by RNase-free water. The quality of the extracted RNA was validated by Bioanalyzer and subjected to sequencing described in (Joseph et al., 2023).

Gene Set Enrichment Analysis (GSEA) (Subramanian et al., 2005) was conducted as detailed in our earlier work (Joseph et al., 2023). Heatmaps and leading-edge enrichment plots were generated using the Molecular Signature Database (MSigDB). One thousand permutations were performed on each gene list tested, and the normalized enrichment score (NES) and nominal P-value were reported for each analysis.

### Proteomic analysis

*3×FLAG-RAB11A-pQXCIP* or *3×FLAG-RAB11B-pQXCIP* was transiently transfected into HEK293T cells. After 24 h, cells were lysed, protein concentrations were measured by Bradford assay, and 2 mg of lysate was immunoprecipitated overnight with anti-FLAG M2 affinity gel beads at 4°C. Proteins were resolved by SDS-PAGE, stained with Ruby Red, and bands ≥25 kDa were analyzed by mass spectrometry. Proteomic analysis of transfected HEK293T cells was described in previous study (Joseph et al., 2023).

### Indirect immunofluorescence microscopy

For immunofluorescence staining, formalin-fixed paraffin-embedded tissue sections were deparaffinized and rehydrated. Antigen retrieval was performed in monohydrate citric acid buffer (pH 6) by boiling slides for 15 min, followed by cooling to 40°C, washing in deionized water for 5 min, and PBS for 5 min. Slides were blocked for 1 h in blocking solution (0.1% Triton-X100, 2% BSA, 2% normal donkey or goat serum in PBS), then incubated overnight at 4°C with primary antibodies diluted in blocking solution. The primary antibodies used included anti-Tom20 (Thermo Fisher, Cat# PA5-52843) which was applied at a 1:500 dilution. After three 10-minute PBS washes, slides were incubated with secondary antibodies diluted in blocking solution for 1 h at room temperature, washed twice in PBS for 10 min each, counterstained with DAPI for 15 min, rinsed in PBS, and mounted using Prolong Gold Antifade (Invitrogen P36930). Imaging was performed using a Zeiss LSM 980 microscope equipped with Airyscan2. Image processing was performed using ImageJ software.

### Live cell imaging

HEK293T cells were grown in Dulbecco’s Modified Eagle’s Medium (DMEM) (Corning, CM10013) supplemented with 10% fetal bovine serum (FBS) (GIBCO, A3160501). Caco2 BBE cells were grown in DMEM, supplemented with 20% FBS. Cells were transiently transfected with mCherry blank, mCherry-tagged RAB11A or RAB11B plasmid using Lipofectamine 3000 (Invitrogen, L3000008). After 12–16 h post-transfection, the cells were incubated with MitoTracker Green (Fisher Scientific, M46750) at a 1:1,000 dilution for 1 h (for HEK293T); 1:1,500 dilution for 15 min (for Caco2 BBE cells). Live cell culture conditions were maintained at 37°C throughout image acquisition with 5% CO2. Single plane confocal or Z-stack imaging were performed using a Zeiss LSM 980 microscope equipped with Airyscan2. 3D Volume Rendering was used to visualize the depth perception of the colocalization, in XY-planar, and Surface Rendering was used to assess the colocalization in XZ-planar. The number of microscopic fields were randomly selected for quantification and was reported in the graph. Number of colocalization points per field were recorded and analyzed with ImageJ, and the statistical result was quantified from at least 3 independent experiments using GraphPad Prism software, employing one-way ANOVA analysis.

### Western blotting

Intestinal tissues were harvested from WT and Rab11b knockout mice. Tissues were lysed in RIPA buffer (Thermo Fisher #89901) with protease inhibitor (Sigma, #11836170001). Total concentration of proteins was determined using the Pierce™ BCA Protein Assay Kit (Thermo Fisher, #23225). 20 μg proteins were resolved on 15% acrylamide-Tris-BIS-0.1% SDS gel, and 6.16% acrylamide-Tris-BIS gel was used as stacking. Proteins were transferred to nitrocellulose membrane (Cytiva, 10600004). Primary antibodies were diluted with 1:1,000 for detection of GAPDH, GeneTex GT239; BCL2, Santa Cruz, sc-7382; NDUFS3, Santa Cruz, sc-374282; SLC25A3, Santa Cruz, sc-376742; and Tom20, Thermo Fisher PA5-52843. Proteins were detected by ECL Prime Western Blotting Detection Reagents (Cytiva, RPN2232).

### Fluorescence-activated cell sorting

The ileum was harvested from each mouse and immediately placed in an ice-cold petri dish containing 1X PBS (Fisher Scientific, SH30256LS). The tissues were cut open longitudinally, and fecal contents were removed by shaking the opened tissue in the PBS-filled petri dish. The tissue was then cut into 5 mm pieces and transferred to a 50 mL Falcon tube containing 30 mL of ice-cold 1X PBS. The pieces were washed by inverting the tube 10–15 times. Washing step was repeated three times, each time transferring the tissue pieces to a new tube with fresh ice-cold 1X PBS. Tissue pieces were transferred to a tube containing 30 mL of crypt isolation buffer, consisting of 1X HBSS without calcium and magnesium (ThermoFisher, 14170112), supplemented with 600 µL of 0.5 M EDTA (Invitrogen, AM9260G). After 15-min, the solution was replaced with fresh crypt isolation buffer, and the tissues were shaken for an additional 30 min at 4°C. The tubes were then vigorously shaken to dislodge epithelial cells, and the turbid solution was passed through a 70 μm cell strainer (Fisher Scientific Cat# 22363548), which was rinsed with 10 mL of PBS that also passed through the cell strainer. Tissue disruption and cell isolation were monitored by brightfield microscopy. The cell suspensions were centrifuged at 500 g for 5 min at 4°C. The resulting pellet was resuspended in 2 mL of TrypLE buffer (ThermoFisher Cat# 12605–028) and split into two 15 mL Falcon tubes, each containing 1 mL of cell suspension in 9 mL of TrypLE buffer. 10 μL of reconstituted DNase (Qiagen Cat#79254) was added to each tube, and the samples were incubated in a 37°C water bath for 20 min, with regular inversions and observations every 3 min to monitor dissociation. When the suspension consisted of greater than 80% single cells, 1 mL of FBS was added to stop the reaction. The solutions were then passed through a 40 μm cell strainer (Fisher Scientific Cat# 22–363–547) into a 50 mL Falcon tube and centrifuged at 700 g for 5 min at 4°C.

For flow analysis of different epithelial cell populations, the cell pellet was resuspended in 500 µL of FACS buffer (F12/DMEM, ThermoFisher, Cat# 12634–010) containing 2 µM ROCK inhibitor (Sigma, Cat# Y0503), 2% BSA, 2% FBS (Sigma, Cat# F2442), and 2 mM EDTA, and then transferred to an Eppendorf tube. The cells were centrifuged at 700 g for 5 min at 4°C and resuspended in 100 µL of FACS buffer containing anti-mouse Fc (1:100, BioLegend, Cat# 101330; RRID: AB_312801) for 10 min on ice. After centrifugation at 700 g for 5 min at 4°C, the pellet was resuspended in 200 µL of a surface staining solution. This solution was prepared by adding EpCAM-APC (CD326) at a 1:200 dilution, CD24-APC/Fire 750 at a 1:200 dilution, and Fc at a 1:100 dilution in FACS buffer. Additionally, DAPI was added at a 1:500 dilution. Alternatively, a different surface staining solution was prepared using CD24-APC/Fire 750 (BioLegend, Cat# 101839) at a 1:200 dilution, EpCAM-FITC (BioLegend, Cat# 118207) at a 1:200 dilution, and Live/Dead 405 (ThermoFisher, Cat# L34963) at a 1:200 dilution.

For MitoTracker and MitoSox analysis, the pellet was resuspended in a staining solution composed of MitoTracker Green (ThermoFisher, Cat# M46750) at a 1:1,000 dilution, MitoSox Red (ThermoFisher, Cat# M36008) at a 1:5,000 dilution, and HBSS with Ca^2+^ and Mg^2+^ (ThermoFisher, Cat# 24020117). The cells were then incubated at 37°C for 30 min. Following the incubation period, 1 mL of FACS buffer was added, and the cells were centrifuged at 700 g for 5 min at 4°C. The resulting cell pellet was resuspended in 500 µL of FACS buffer, and 1 µL of DAPI was added before flow cytometry sorting. Alternatively, the cells were incubated at 37°C for 30 min with MitoTracker Orange (Invitrogen, Cat# M7510) at a 1:1,000 dilution. Cells were then sorted using the BD Biosciences Aria II Flow Cytometer (BD FACSAria II). For all fluorophores, a cell pellet was stained with a single antibody corresponding to each primary antibody used, and a combination of all antibodies was utilized to establish compensation controls. Single viable epithelial cells, Paneth cells, and stem cells were gated based on forward scatter, side scatter, and negative staining for DAPI or Live/Dead marker. Subpopulations were further gated using EpCAM, CD24, MitoTracker, or MitoSox CellROX.

### Quantification and statistical analysis

Immunostaining and live cell imaging results were quantified on 10 to 12 independent microscopic fields and imaged using a ZEISS LSM 980 microscope. All flow cytometry statistical data was obtained using FlowJo software (TreeStar, version 10.7.1). Statistical analyses were performed using GraphPad Prism software, employing unpaired t-tests for two-group comparisons and one-way ANOVA for multigroup analysis.

### Data availability

The RNA‐Seq datasets are available in Gene Expression Omnibus, accession number: GSE232493 (https://www.ncbi.nlm.nih.gov/geo/query/acc.cgi?acc=GSE232493). The mass spectrometry proteomics data have been deposited to the ProteomeXchange Consortium via the PRIDE partner repository with the dataset identifier PXD042300 and PXD042335.

## Results and discussion

### Proteomic analysis reveals RAB11B association with mitochondrial proteins

**FIGURE 1:**
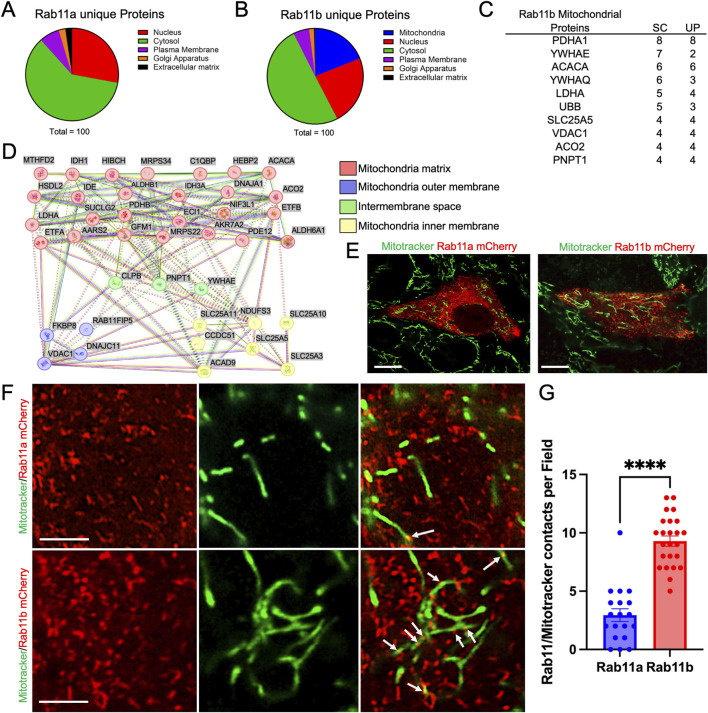
**(A, B)** Proteomic analysis of RAB11A and RAB11B unique proteins classified into different cellular compartments: Nucleus, Cytosol, Plasma Membrane, Golgi Apparatus, and Extracellular Matrix. **(C)** The top 10 RAB11B mitochondrial-related proteins ranked by number of spectrum counts (SC) from high to low. Unique peptide (UP) numbers are also provided for each target. **(D)** STRING network analysis of *Rab11b* co-precipitated mitochondrial-related proteins, categorized by their localization within mitochondrial substructures, including the matrix, outer membrane, intermembrane space, and inner membrane. **(E, F)** Live cell imaging of HEK293T cells transfected with mCherry-tagged wild-type Rab11a and *Rab11b*. White arrows indicate points of contact between *Rab11* and mitochondria. Scale bar: 10 μm in E; 1 μm in F. **(G)** Quantification of RAB11 and mitochondrial contacts per field in cells expressing mCherry-tagged RAB11A or RAB11B. The data points represent 18 and 24 individual fields from 3 independent experiments.

To profile the protein interactome associated with RAB11A or RAB11B, we conducted proteomic analyses employing mass spectrometry on 3× Flag-tagged RAB11A and RAB11B co-immunoprecipitants from HEK293T cells (Joseph et al., 2023). The mass spectrometry data was sorted to identify interacting partners that were unique to RAB11A but not RAB11B (Figure 1A) and interactors unique to RAB11B but not RAB11A (Figure 1B). Notably, the RAB11B proteomic network revealed a significant association with mitochondrial proteins, including the RAB11FIP5 (RAB11 family interacting protein 5) (Figures 1C,D). The top 10 mitochondrial protein targets, ranked by spectrum counts and unique peptide numbers, in RAB11B-mitochondria network were proteins involved in metabolism and mitochondrial regulation (Figure 1C). String analysis of these RAB11B mitochondrial proteome were strongly related to the mitochondrial matrix (Figure 1D).

**FIGURE 2:**
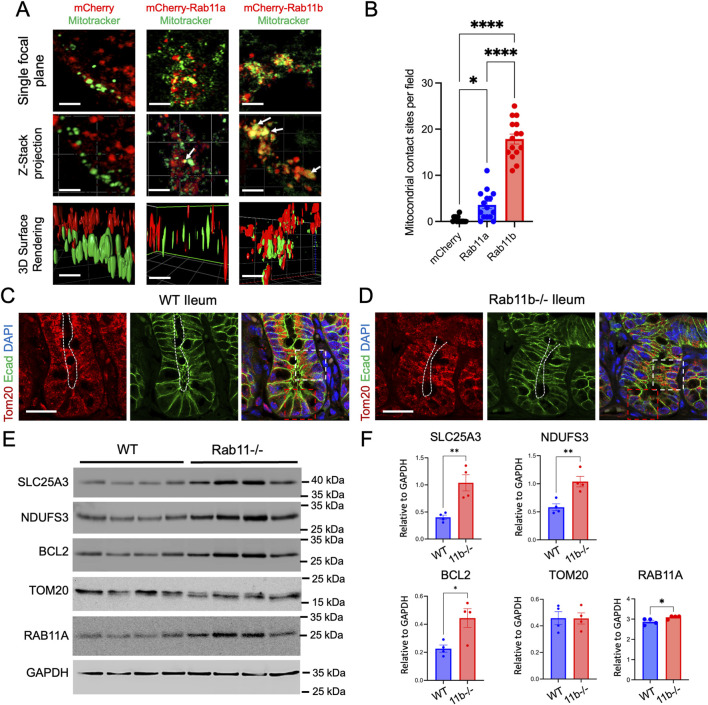
**(A)** Live cell imaging of Caco2 BBE cells transfected with mCherry, mCherry-tagged wild-type Rab11a or *Rab11b*. White arrows indicate points of contact between *Rab11* and mitochondria. Scale bar: 2 μm. **(B)** Quantification of mCherry and mitochondrial contacts per field in cells. Data points represent 15 fields each condition from 3 independent experiments. **(C–D)** Immunofluorescence staining of WT and Rab11b KO ileum tissues, showing Tom20 (red), E-cadherin (green), and DAPI (blue). White and red boxes indicate transit amplifying and crypt regions, respectively. White dotted lines indicate the luminal (apical) surface. Scale bar: 50 μm. **(E)** Western blot analysis of specific mitochondrial proteins in WT and Rab11b intestinal tissues. **(F)** Quantification of Western blots.

To appreciate the cellular localization of RAB11A, RAB11B, and potential intersection with mitochondria, HEK293T cells were transiently transfected with either mCherry-tagged RAB11B or RAB11A and subjected to live cell fluorescence confocal microscopy (Figure 1E). The RAB11B and RAB11A fluorescent signals appeared to be structures that are typically identified as vesicular puncta (Figure 1E). In comparison of MitoTrackerGreen and RAB11 A/B fluorescence signals, there were distinct contacting “spots” where the fluorescence signals for mitochondria and RAB11B appear to be co-localized at this level of resolution (see Figure 1F). Such colocalization spots were more readily observed with RAB11B as compared to RAB11A in experiments conducted in parallel (Figure 1G). We performed similar live cell imaging experiments in Caco2 cells to determine if the observation holds in this intestinal epithelial cell line where endogenous RAB11 is abundant. Single plane confocal, Z-stack imaging and 3D surface rendering revealed RAB11-mitochondrial contact sites in Caco2 cells (Figure 2A), while RAB11B has significantly more of these contact sites than RAB11A or mCherry alone (Figure 2B).

To examine if there is general mitochondrial defect *in vivo*, we performed immunostaining for Tom20, a translocase of outer mitochondrial membrane, in wild type (WT) and *Rab11b* knockout (KO) intestinal tissues. Tom20 localization was observed throughout the entire cell, with prominent staining observed in the subapical cytoplasm region (white box in Figure 2C). In contrast, Tom20 staining appeared to be disrupted in the Rab11b KO epithelial cells and became localized to the entire cell body in the crypt-villus junction region (white box in Figure 2D). The signal became weaker in Rab11b KO crypt cells (red box in Figure 2D).

We then performed Western Blotting analysis to assess the mitochondrial protein abundances in WT and Rab11b KO intestinal tissues. While the total Tom20 abundances remained unchanged, SLC25A3, a mitochondrial phosphate carrier, and NDUFS3, a vital part of mitochondrial complex I of the respiratory chain, were both increased in Rab11b KO intestines (Figures 2E,F). Interestingly, BCL2, a key regulator of the permeabilization of the outer mitochondrial membrane in programmed cell death, was also increased in Rab11b KO intestinal tissues (Figures 2E,F). These biochemical and immunohistochemical results suggest that mitochondrial localizations and functions are altered in the absence of Rab11b.

### *Rab11b*-deficient mouse intestines have altered mitochondrial transcriptomics

**FIGURE 3:**
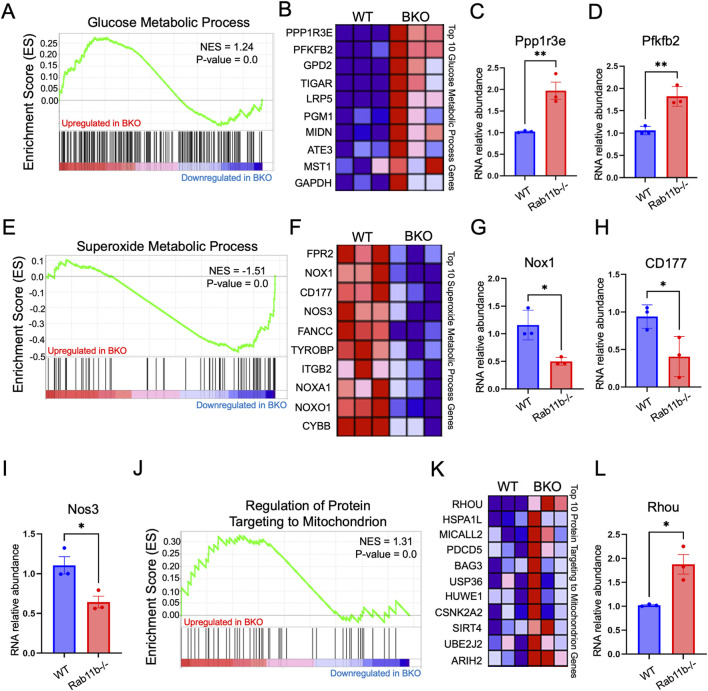
**(A)** Gene Set Enrichment Analysis (GSEA) of bulk RNA sequencing data targeting the glucose metabolic process transcriptome, comparing WT and BKO mice. *P*‐value <0.001 (n = 3 mice per genotype). **(B)** Heat map displaying the expression of glucose metabolic process pathway genes in WT and RAB11BKO intestinal transcriptomes. *P*‐value <0.001 (n = 3 per genotype). **(C, D)** Relative RNA abundance of glucose metabolic process genes, Ppp1r3e and Pfkfb2. *P*‐value <0.001 (n = 3 mice per genotype). **(E)** GSEA of the superoxide metabolic process transcriptome in WT and BKO intestinal samples. *P*‐value <0.001 (n = 3 per genotype). **(F)** Heat map showing superoxide metabolic process genes in WT and RAB11BKO intestinal transcriptomes *P*‐value < 0.001 (n = 3 per genotype). **(G–I)** Relative RNA abundance of Nox1, CD177, and Nos3 in WT and RAB11BKO intestinal samples *P*‐value < 0.001 (n = 3 per genotype). **(J)** GSEA of the “Regulation of Protein Targeting to Mitochondrion” transcriptome comparing WT and RAB11BKO. *P*‐value <0.001 (n = 3 per genotype) **(K)** Heat map showing RNA expression levels of key genes involved in protein targeting to mitochondria in WT and RAB11BKO intestinal transcriptomes (n = 3 per genotype) *P*‐value < 0.001 (n = 3 per genotype). **(L)** Relative RNA abundance of Rhou in WT and RAB11b KO tissues. *P*‐value <0.001 (n = 3 per genotype).

To examine if *Rab11b* deletion impacted the intestinal transcriptomic readouts of mitochondrial function, we analyzed the intestinal bulk-RNA sequencing from WT and *Rab11b* KO littermates. Gene set enrichment and heat map analysis revealed a significant increase of glucose metabolism in *Rab11b* KO intestines compared to WT (p < 0.05, Figures 3A,B). Specifically, PPP1R3E that is involved in positive regulation of glycogen biosynthetic process and PFKFB22 that regulates the level of F26BP for control of glycolysis process were robustly increased in *Rab11b* KO intestines (Figures 3C,D). Interestingly, genes related to superoxide metabolic processes are reduced in *Rab11b* KO intestinal tissue (Figures 3E,F). Reductions in genes such as CD177, FANCC, TYROBP and ITGB2 (Figures 3F–I), which regulate inflammation and superoxide production, suggest that *Rab11b* ablation potentially disrupted the trafficking of these receptors and mitochondrial superoxide production.

Gene set enrichment analysis identified that genes related to mitochondrial protein targeting are increased in RAB11B KO intestines (Figure 3J). Figure 3K provides heat maps for the top 10 hits and three of these, HSPA1L, PDCD5 and BAG3, are linked to maintaining mitochondrial homeostasis and integrity (Tahrir et al., 2017; Bock et al., 2015; Ni et al. 215). Interestingly, RhoU GTPase is significantly elevated in *Rab11b* KO tissues (Figure 3L) (Hall, 1998) and this Rho family member appears to play a role in epithelial cell development, actin cytoskeletal dynamics, and motor protein activity. Overall, these results suggest that genetic ablation of *Rab11b* led to an enhanced mitochondrial protein targeting gene signature possibly in response to an altered mitochondrial function in tissue homeostasis.

### *Rab11b* deficient enterocytes have impaired mitochondrial structure and function

**FIGURE 4:**
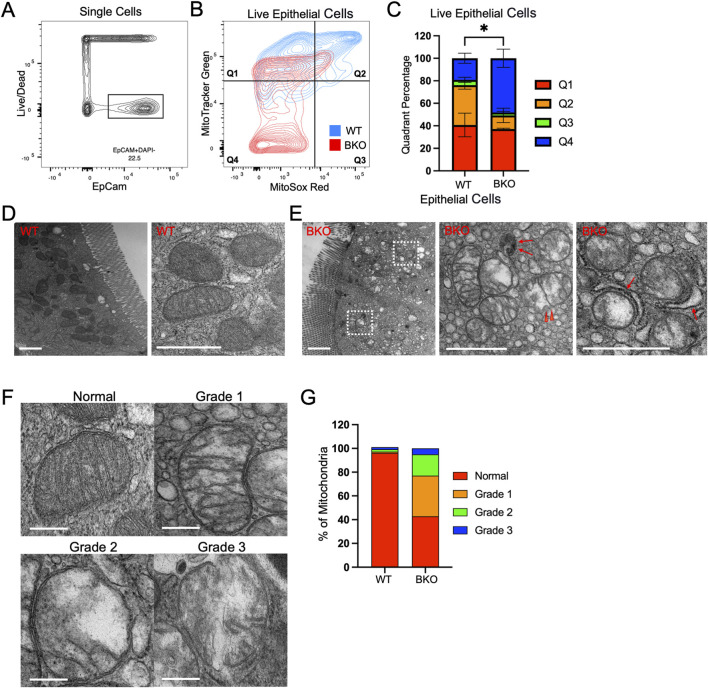
**(A)** Total epithelial single cells isolated from WT and Rab11b KO mice, gated using EpCam (x-axis) and Live/Dead (y-axis) markers. **(B)** Flow cytometric analysis of live epithelial cells stained with MitoTracker Green and MitoSOX Red, divided into four quadrants. WT (blue) and Rab11b KO (red) cells are shown. **(C)** Quantification of the percentage of cells in each quadrant (Q1-Q4) for WT and Rab11b KO samples. **(D)** Representative electron microscopy images of WT mitochondria showing intact cristae and regular structure. Scale bars: 1 μm. **(E)** Electron microscopy images of Rab11b KO mitochondria displaying abnormal morphology, including swelling and loss of cristae integrity. Arrowheads point to discontinued outer membrane. Red arrows point to potential autophagic vesicles and mitophagy events. Scale bars: 1 μm. **(F)** Representative electron microscopy images showing the progression of mitochondrial damage, graded from normal to Grade 3, with increasing disruption. Scale bars: 0.5 μm. **(G)** Quantitative analysis of mitochondrial grading, comparing the percentage distribution of normal and damaged mitochondria between WT and Rab11b KO cells. Data represent 50–70 microscopic images from 3 independent samples per genotype.

To examine if *Rab11b* was required for mitochondrial activity, we used fluorescence-activated cell sorting (FACS) of intestinal epithelial cells isolated from WT and *Rab11b* KO crypts prepared from small intestine. The fluorescent probes MitoTracker Green (green fluorescence) and MitoSox (red fluorescence) were used to measure mitochondrial quantity and superoxide for mitochondrial activity, respectively. EpCAM^+^DAPI^−^ cells were used to gate for live epithelial cells (Figure 4A). When gating for MitoTracker Green/MitoSox signals, live epithelial cells with the highest mitochondrial number and mitochondrial activity were positioned in Quadrant 2 (Figure 4B). Notably, the number of *Rab11b* KO epithelial cells in Quadrant 2 were significantly reduced relative to the number of WT (Figures 4B,C). *Rab11b* KO epithelial cells were most abundant in Quadrant 4 that represented cells with low mitochondrial content and activity (Figures 4B,C). These results echo the transcriptomic results indicating an altered mitochondrial function upon *Rab11b* deletion.

By transmission electron microscopy (EM), WT enterocytes, readily identified by their brush borders, contained abundant mitochondria with well-defined inner and outer membranes, transverse cristae, and electron dense matrix. Consistent with fluorescence localization (Figure 2C), EM revealed that mitochondria were largely positioned in the apical domain of WT enterocytes (Figure 4D). *Rab11b* knockout enterocytes still elaborated apical brush borders, however their mitochondria lacked organization and electron density seen for WT mitochondria (Figure 4E). Mitochondria in *Rab11b* KO epithelial cells showed a dramatic reduction in cristae, associated with breakage and loss of transverse cristae, loss of matrix electron density, and discontinuous outer membrane in some cases (red arrowheads in Figure 4E). Events of mitophagy or autophagosomes appeared more visible in *Rab11b* KO cells (arrows in Figure 4E), potentially reflecting an early reported observation in HeLa cells (Grimsey et al., 2016). Mitochondrial structural integrity was categorized into mild (grade 1), moderate (grade 2) or severe (grade 3) loss of integrity (Figure 4F). *Rab11b* KO enterocytes showed an overall 60% reduction in normal-appearing mitochondria, with over 20% mitochondria exhibiting moderate to severe structural or membrane damage (Figure 4G). These ultrastructural findings were validated in different KO mice that were analyzed in parallel with WT mice, confirming that *Rab11b* is required for maintaining mitochondrial morphology in enterocytes.

### *Rab11b* is required for mitochondrial functional integrity in intestinal epithelial cells

**FIGURE 5:**
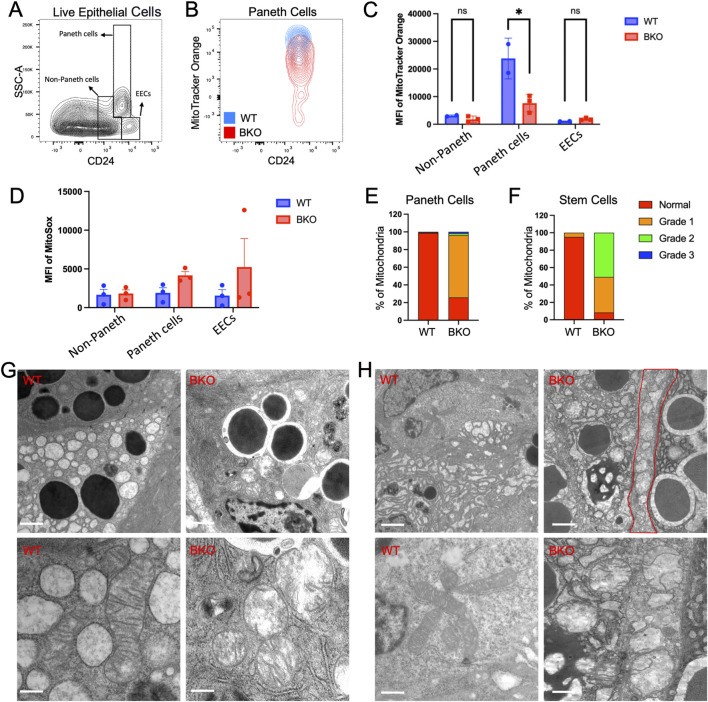
**(A)** Gating strategy for identifying Paneth cells, non-Paneth cells, and enteroendocrine cells (EECs) based on side scatter (SSC-A) and CD24 expression. **(B)** Flow cytometry contour plots showing MitoTracker Orange intensity in Paneth cells from WT (blue) and BKO (red) mice. **(C, D)** Quantification of MitoTracker Orange and mean fluorescence intensity (MFI) of MitoSOX in non-Paneth cells, Paneth cells, and EECs. **(E, F)** Mitochondrial morphology grading in Paneth cells and stem cells illustrates normal and damaged mitochondria distribution in WT and BKO samples. **(G)** Representative electron microscopy images show mitochondria’s morphological differences between WT and BKO Paneth cells. **(H)** Comparison of mitochondrial structure in non-Paneth cells and enteroendocrine cells (EECs) between WT and BKO mice. Data represent 50–70 microscopic images from 3 independent mouse tissue samples per genotype.

To examine if the observed mitochondrial defects were also present in non-absorptive cells, such as intestinal stem cells and Paneth cells in crypts, we performed additional flow cytometry analysis using gating strategy (Sato et al., 2011), specific for CD24^+^ non-Paneth cells, Paneth cells, and enteroendocrine cells (EECs) (Figure 5A). Mitotracker Orange (CMTMRos), a cationic fluorescent probe for negative mitochondrial membrane potential, showed that WT Paneth cells have a relatively higher signal accumulation than Rab11b KO Paneth cells, indicating a reduced mitochondrial activity in the absence of Rab11b (Figure 5B). This reduction was not observed in non-Paneth crypt epithelial cells (Figure 5C). However, mitochondrial superoxide labeled by MitoSox Red did not significantly change or showed an insignificant increase in *Rab11b* KO Paneth cells (Figure 5D), which could potentially result from electron leakage from damaged mitochondria. These results collectively suggest that mitochondrial membrane potential and metabolic activity are changed in response to *Rab11b* loss, with Paneth cells potentially being more vulnerable.

Electron microscopy of mitochondria in Paneth (Figure 5G) and stem cells (Figure 5H) confirmed the disrupted mitochondrial inner membranes and cristae structures described in *Rab11b* KO enterocytes. Quantitative analysis of mitochondrial structure revealed that approximately 80%–90% mitochondria in *Rab11b* deficient Paneth cells and stem cells are abnormal (Figures 5E,F). Thus, the loss of electron density within the mitochondrial matrix induced by *Rab11b* loss appear to be consistent across different intestinal epithelial cell types. These findings collectively suggest that *Rab11b* is crucial for maintaining mitochondrial structure and function in gut epithelial cells.

Several prior studies have indicated that Rab proteins may play a role in regulating mitochondrial dynamics. For instance, it has been shown that depolarized mitochondria can be sequestered in Rab5-positive endosomes, which later mature into Rab7-positive late endosomes before being delivered to lysosomes for degradation (Hammerling et al., 2017). Furthermore, Rab8a has been identified as a mitochondrial receptor for lipid droplets (Ouyang et al., 2023), while Rab32 has been shown to coordinate mitochondrial fission (Alto et al., 2002).

Notably, RAB11A, which primarily localizes to the recycling endosomal compartment, appears to regulate the mitochondrial recruitment of the fission protein Drp1 through its effector FIP1C/RCP, which ultimately modulates mitochondrial fission and fusion events (Landry et al., 2014). However, the role of the closely related small GTPase RAB11B in mitochondrial function remains largely unexplored. Our collective observations from *Rab11b* genetic ablation and ultrastructural characterization confirmed a distinct mitochondrial defect in different IEC cell types. Our proteomic and live cell imaging analysis pointed to a potential physical intersection of *Rab11b* vesicles with mitochondrial protein complex. Loss of *Rab11b* may affect mitochondrial movement, assembly or fission, as reflected by the drastic disruption of mitochondrial membrane integrity and functions related to membrane potential and ROS production. The transcriptomic results from *Rab11b* knockout intestines suggested a potential tissue compensation in response to the impaired mitochondrial functions. Taken together, we propose a previously unappreciated contribution of RAB11B to the maintenance of mitochondrial structure and homeostasis in the gut epithelial cells.

## Data Availability

The original contributions presented in the study are included in the article/supplementary material, further inquiries can be directed to the corresponding author.
